# Application of radiomics-based multiomics combinations in the tumor microenvironment and cancer prognosis

**DOI:** 10.1186/s12967-023-04437-4

**Published:** 2023-09-06

**Authors:** Wendi Kang, Xiang Qiu, Yingen Luo, Jianwei Luo, Yang Liu, Junqing Xi, Xiao Li, Zhengqiang Yang

**Affiliations:** 1 Department of Interventional Therapy, National Cancer Center/National Clinical Research Center for Cancer/Cancer Hospital, Chinese Academy of Medical Sciences and Peking Union Medical College, Panjiayuan Nanli 17# Chaoyang District, Beijing, 100021 China; 2 Obstetrics and Gynecology Hospital of, Fudan University, Shanghai, 200011 China; 3 Department of Diagnostic Radiology, Hunan Cancer Hospital and the Affiliated Cancer Hospital of Xiangya School of Medicine, Central South University, 283 Tongzipo Road, Yuelu District, Changsha, 410013 Hunan China; 4 Department of Thoracic Surgery, National Cancer Center/National Clinical Research Center for Cancer/Cancer Hospital, Chinese Academy of Medical Sciences and Peking Union Medical College, Beijing, China

**Keywords:** Multiomics combination, Radiomics, Biomarkers, Tumor microenvironment, Cancer prognosis

## Abstract

The advent of immunotherapy, a groundbreaking advancement in cancer treatment, has given rise to the prominence of the tumor microenvironment (TME) as a critical area of research. The clinical implications of an improved understanding of the TME are significant and far-reaching. Radiomics has been increasingly utilized in the comprehensive assessment of the TME and cancer prognosis. Similarly, the advancement of pathomics, which is based on pathological images, can offer additional insights into the panoramic view and microscopic information of tumors. The combination of pathomics and radiomics has revolutionized the concept of a “digital biopsy”. As genomics and transcriptomics continue to evolve, integrating radiomics with genomic and transcriptomic datasets can offer further insights into tumor and microenvironment heterogeneity and establish correlations with biological significance. Therefore, the synergistic analysis of digital image features (radiomics, pathomics) and genetic phenotypes (genomics) can comprehensively decode and characterize the heterogeneity of the TME as well as predict cancer prognosis. This review presents a comprehensive summary of the research on important radiomics biomarkers for predicting the TME, emphasizing the interplay between radiomics, genomics, transcriptomics, and pathomics, as well as the application of multiomics in decoding the TME and predicting cancer prognosis. Finally, we discuss the challenges and opportunities in multiomics research. In conclusion, this review highlights the crucial role of radiomics and multiomics associations in the assessment of the TME and cancer prognosis. The combined analysis of radiomics, pathomics, genomics, and transcriptomics is a promising research direction with substantial research significance and value for comprehensive TME evaluation and cancer prognosis assessment.

## Introduction

**Fig. 1:**
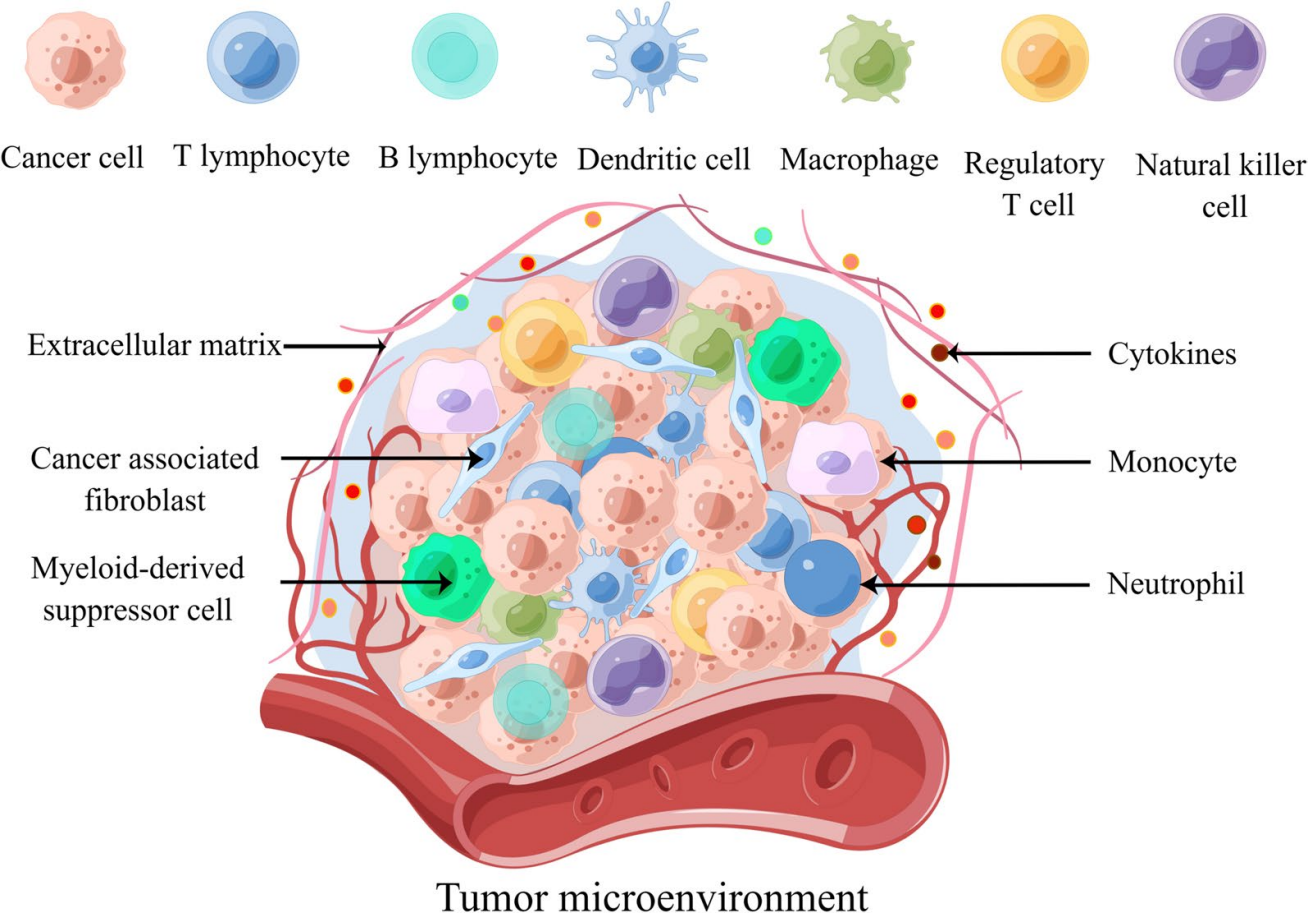
Schematic diagram of the various components of the tumor microenvironment, which contains multiple cell types and molecules

The tumor microenvironment (TME) is a multicellular ecosystem composed of multiple cell types and molecules that is highly dynamic and spatially heterogeneous over time [1, 2] (Fig. 1). The TME plays a crucial role in promoting and sustaining tumor characteristics by releasing important molecules and activating related signaling pathways. These pathways interact with tumor cells, influencing their plasticity, invasiveness, and migratory capacity. Conversely, the shaping of the TME by tumor cells can also affect the biological properties of the tumor itself [3–6]. The functional diversity and temporal sequence of the components of the TME are subject to dynamic changes under the influence of other components, which collectively impact tumor development and have significant clinical implications for treatment and prognosis [7–9].

Immunotherapy has revolutionized cancer treatment [10, 11]. However, despite its potential, the objective response rate of immunotherapy in most cancers remains relatively low [12–14]. Therefore, there is an urgent need to identify reliable biomarkers that can screen patients who are most likely to benefit from immunotherapy, thereby guiding its use more effectively and rationally. Tumor-infiltrating lymphocytes (TILs) are one of the most representative biomarkers [15, 16] and have shown good prognostic predictive power in several cancer types [17–19]. Recently, tertiary lymphoid structures have also been used as significant predictive biomarkers. Their presence is associated with a good prognosis and response to immunotherapy and may activate antitumor immune responses [20–23]. However, assessment of these important biomarkers still relies mainly on pathological methods. The invasive nature of pathological tests and the limitations and biases in the acquisition of tumor specimens hinder the widespread use of the TME and prognostic assessment. Therefore, there is an urgent need to develop new noninvasive assessment methods to facilitate comprehensive decoding of the TME and prediction of cancer prognosis.

Medical imaging provides a wealth of information that reflects not only the macroscopic anatomy of the entire tumor but also its microscopic heterogeneity and the functional status of the surrounding environment [24, 25]. Quantitative analysis of imaging data can offer a more comprehensive panoramic view of the tumor than examining a small portion of tumor tissue pathologically [26]. Radiomics is a rapidly growing research area focused on the conversion of images of the region of interest into quantitative radiomics features. These features are derived through high-throughput extraction of information from imaging data, enabling their combination with the clinicopathological features of patients and subsequent modeling using machine learning algorithms [27]. Radiomics features encompass a range of characteristics, including shape features such as volume, first-order features such as skewness, second-order features such as gray level co-occurring matrix, and higher-order features such as Wavelet [28]. The essence of radiomics lies in the efficient extraction of quantitative image features to effectively characterize the lesion region of interest. These radiomics features capture tissue and lesion characteristics that, in conjunction with clinical, laboratory, histopathology, genomics, or other histological data, can be integrated through machine learning. Their primary utility lies in addressing diverse clinical problems, including accurate diagnosis, efficacy assessment, and prognosis prediction of diseases [29]. The radiomics process mainly includes four fundamental steps: image acquisition, segmentation, feature extraction, and model building [30]. Image segmentation methods encompass manual segmentation, semiautomatic segmentation, and fully automatic segmentation. A growing number of radiomics studies have confirmed the correlation between radiomics features and TME components. However, due to the highly dynamic and temporal-spatial heterogeneity of the TME, a single radiomic approach for evaluating the TME and cancer prognosis may be limited. With the development of genomics and pathomics, multiomics combinations offer new paths and directions for the assessment of the TME and cancer prognosis.

This review provides a comprehensive summary of the studies of important biomarkers in radiomics for assessing the TME, highlighting the interconnection between radiomics, genomics, and pathomics in cancer. We aimed to comprehensively decode the TME and predict cancer prognosis by multiomics combinations, which has important research significance and clinical value. In the future, further research is needed to address the challenges of multiomics analysis and to fully realize the potential of integrating different types of data to improve our understanding of the TME and its role in cancer.

## Radiomics predicts important components of the tumor microenvironment

Previous research in oncology has primarily concentrated on studying tumor cells, disregarding the crucial role of the TME in tumorigenesis and disease progression. TME research holds clinical significance because it enables the prediction of treatment effectiveness and the design of targeted therapeutic strategies, among other essential aspects. Further improvements in current immunotherapies or the development of novel therapeutic approaches greatly depend on understanding the interactions and immune evasion mechanisms of the various components within the TME. Accurate characterization of the specific components and features of the TME is vital for evaluating tumor prognosis, enhancing clinical decision-making, and advancing precision medicine.

The composition of the TME varies depending on the relative proportions of its different constituents, while its presence plays a critical role in tumor growth and invasion. When immune cells are lacking in the TME or when immunosuppressive cells are excessively present, the efficacy of anti-programmed cell death protein-1 (PD-1) and programmed cell death ligand-1 (PD-L1) immunotherapy is diminished. Notably, the TME significantly influences the survival benefits of immunotherapy [31]. The presence of immune cells within the TME, including the percentage of CD8^+^ T cells, can serve as a predictor of immunotherapy effectiveness [32]. Therefore, enhancing the population of immune cells is a promising approach to enhance the efficacy of tumor treatment. Immune cells within the TME exhibit a dualistic function—recognizing and destroying tumor cells on the one hand, while also facilitating tumor growth and metastasis on the other hand [33, 34]. For instance, immune cells, including T cells, B cells, macrophages, and myeloid-derived suppressor cells (MDSCs), possess the ability to modify the TME, thereby impacting the metastatic and pathological traits of tumors. Tumor-associated macrophages can facilitate angiogenesis and invasion through the secretion of cytokines, growth factors, and proteases [35]. Activated tumor-associated fibroblasts have the capability to secrete various substances, including extracellular matrix and vascular endothelial growth factor (VEGF), among others, thereby contributing to the complex nature of the TME [36]. The extracellular matrix can exert influence over cell growth, metastasis, and the evasion of immune surveillance through the activation of signaling pathways, which subsequently impacts the pathogenesis of tumors. Additionally, tumor cells have the ability to release several growth factors, such as tumor growth factor and endothelial growth factor, as well as VEGF, which serve to enhance the development of new blood vessels [37]. Neovascularization is critical in providing nourishment and oxygen to tumor cells, ultimately playing a pivotal role in tumor growth.

In conclusion, the TME plays a crucial role in tumor growth and metastasis. Gaining a comprehensive understanding of TME formation, investigating the crosstalk between immune cells and tumors, and exploring multiple genetic variants are future directions of TME research. Additionally, selecting targeted therapeutic strategies based on TME typing can enhance the effectiveness of tumor treatment. Accurately predicting and classifying significant TME components are essential for targeted tumor therapy and prognostic assessments. This section aims to summarize ongoing efforts in predicting and evaluating relevant TME components and cancer prognosis through radiomics.

### Tumor-infiltrating lymphocytes

TILs represent a vital constituent of the TME and are involved in the local immune response to the TME. Previous studies have shown that the presence, differentiation and localization of TILs determine clinical outcomes, prognostic assessments and clinical decision making [38, 39]. However, specimens used for TIL assessment are not representative of TIL levels in the whole tumor due to tumor heterogeneity and differences and limitations in pathology specimen acquisition. Therefore, preoperative assessment and prediction of TIL levels by noninvasive radiomics methods are important. Table 1 summarizes the studies related to radiomics prediction of TILs.

**Table 1:** Radiomics study for predictive evaluation of tumor-infiltrating lymphocytes

Tumor type	Sample size	TILs evaluation	TILs evaluation method	Imaging modality	Feature selection	Model	Training set performance	Validation set performance	References
N/A	254	CD8^+^TILs	RNA-seq	CT	Elastic-net	Linear regression	AUC 0.74	AUC 0.67	[43]
UPS	14	CD8^+^TILs	RNA-seq	MRI	N/A	N/A	ACC 93%	N/A	[54]
TNBC	139	TILs	RNA-seq	MRI	Elastic net	LR	AUC 0.868	AUC 0.790	[46]
TNBC	43	TILs	IHC	Mammography	Mann–Whitney U-test/PCC	N/A	N/A	N/A	[55]
TNBC	80	TILs	HE	MRI	N/A	N/A	AUC 0.752	N/A	[51]
BC	133	TILs	HE	MRI	LASSO	LR	AUC 0.934	AUC 0.872	[47]
BC	172	TILs	IHC	MRI	LASSO	Linear regression	AUC 0.742	AUC 0.718	[56]
BC	121	TILs	HE	Mammography	RFE	LR	AUC 0.83	AUC 0.79	[57]
BC	154	TILs	NA	MRI	LASS0	LR	AUC 0.86	AUC 0.83	[58]
RC	141	CD8^+^TILs	IHC	MRI	LASSO	Linear regression	AUC 0.760	AUC 0.729	[42]
RC	133	T cells	IHC	MRI	GBDT	LR	AUC 0.770	AUC 0.768	[59]
CCLM	103	T cells	IHC	CT	N/A	SVM	N/A	N/A	[60]
HGG	51	T cells	FCM	MRI	sPLS	sPLS-DA	AUC 0.986	N/A	[61]
LGG	107	B/T cells	RNA-seq	MRI	LASSO	COX	R correlation coefficient		[62]
							0.975 (B cell)	0.429	
							0.474 (CD8 T cell)	0.552	
NSCLC	100	TILs	IHC	CT	Mann–Whitney U	Cox model	AUC 0.91	N/A	[52]
NSCLC	103	CD8^+^TILs	IHC	PET/CT	LASSO	LR	AUC 0.800	AUC 0.794	[63]
NSCLC	290	TILs	IHC	CT	N/A	COX	N/A	N/A	[64]
NSCLC	117	CD8^+^TILs	IHC	CT	LASSO	N/A	AUC 0.83	AUC 0.68	[65]
NSCLC	60	TILs	IHC	CT	PCA	N/A	N/A	N/A	[66]
NSCLC	149	CD8^+^T cells	RNA-seq	CT	N/A	RF	AUC 0.681 (RF)	N/A	[67]
						LDA	AUC 0.674 (LDA)		
						CART	AUC 0.647 (CART)		
NSCLC	97	CD8^+^TILs	FACS	CT	N/A	Neural network	0.788	0.753	[68]
NSCLC	91	CD8^+^TILs	IHC	PET-CT	LASSO	LR	0.818	N/A	[69]
NSCLC	44	CD8^+^TILs	IHC	PET	N/A	COX	0.9	N/A	[70]
NSCLC	221	CD8^+^T cells	IHC	PET/CT	LASSO	LR	0.907	0.883	[71]
PDAC	184	CD8^+^TILs	IHC	CT	LASSO	XGBoost	AUC 0.75	AUC 0.67	[48]
PDAC	156	CD20B cells	IHC	MRI	LASSO	LR	AUC 0.79	AUC 0.79	[72]
PDAC	114	CD8^+^T cells	IHC	MRI	LASSO	LDA	AUC 0.85	AUC 0.76	[73]
PDAC	183	TILs	IHC	CT	NA	XGBoost	AUC 0.93	AUC 0.79	[74]
PDAC	156	TILs	IHC	MRI	LASSO	XGBoost	AUC 0.86	AUC 0.79	[49]
HCC	142	CD8^+^T cells	IHC	CT	Elastic-net	linear regression	AUC 0.751	0.705	[50]
HCC	207	T cells	IHC	MRI	Randomized tree	Randomized tree	AUC 0.904	AUC 0.823	[75]
HNSCC	160	CD8^+^T cells	RNA-seq	CT	Consensus clustering	RF	ACC 65.7%	N/A	[76]
HNSCC	71	CD8^+^T cells	IHC	CT	LASSO	LR	0.786	N/A	[77]
ESCC	220	CD8^+^T cells	IHC	CT	LASSO	LR	0.764	0.728	[78]

*UPS* undifferentiated pleomorphic sarcomas, *HGG* high-grade gliomas, *TNBC* triple-negative breast cancer, *BC* breast cancer, *NSCLC* non-small cell lung cancer, *PDAC* pancreatic ductal adenocarcinoma, *HCC* hepatocellular carcinoma, *LGG* lower-grade gliomas, *CCLM* colorectal cancer lung metastasis, *HNSCC* head and neck squamous cell carcinoma, *ESCC* esophageal squamous cell carcinoma, *CART* classification and regression tree, *H&E* hematoxylin and eosin, *IHC* immunohistochemistry, *PET* Positron emission tomography, *LASSO* least absolute shrinkage and selection operator, *ACC* accuracy, *sPLS-DA* sparse partial least squares discriminant analysis, *RFE* recursive feature elimination, *LR* logistic regression, *GBDT* gradient boosting decision tree, *PCC* Pearson correlation coefficient, *PCA* principal component analysis, *RNA-seq* RNA-sequencing, *LDA* linear discriminative analysis, *SVM* support vector machine, *RF* random forest, *FACS* fluorescence-activated cell sorting, *FCM* flow cytometry, *AUC* area under the curve, *N/A* not applicable

Recently, radiomics studies concerning TILs have mainly focused on breast cancer, lung cancer, and pancreatic ductal adenocarcinoma. A small number of studies have also included hepatocellular carcinoma, colorectal cancer, glioma, head and neck squamous cell carcinoma, esophageal cancer, and undifferentiated pleomorphic sarcoma. The vast majority of these studies have demonstrated good predictive performance. Radiomics studies of TILs encompass three main aspects. (1) Most studies have focused on the use of radiomics for preoperative prediction of TIL level and density. (2) A few studies have combined radiomics features with TILs to predict prognosis. (3) The TME has been defined as “cold” or “hot” based on the abundance of TILs combined with PD-L1/PD-1 expression in tumors for the prediction and exploration of relevant TME immune features.

Radiomic assessment of the TME first began with prediction of the density of a particular immune cell. Braman et al. showed that classifiers of intratumoral and peritumoral magnetic resonance imaging (MRI) radiomic features predicted the breast cancer HER2-E subtype and that features in the peritumoral 0–3 mm region correlated significantly with the density of TILs [40]. Khorrami et al. demonstrated an association between computed tomography (CT) radiomics-based peritumoral Gabor features and TIL density in response to immunotherapy in lung cancer patients [41]. Jeon et al. found a significant correlation between MRI radiomic features and CD8^+^ TIL density when predicting alterations in CD8^+^ TIL density induced by radiotherapy in locally progressive rectal cancer [42].

A study was conducted by Sun et al. to forecast the TIL level using radiomics. They effectively assessed tumor-infiltrating CD8 cells and immunotherapeutic response by CT radiomics in a variety of advanced solid tumors, and the radiomic signature of CD8 cells was validated in three additional independent cohorts, providing precise predictions for distinguishing the immunophenotypes of tumors and clinical outcomes of cancer patients undergoing anti-PD-1 and PD-L1 immunotherapy [43]. Sun et al. used validated CD8 cell radiomic features to predict the prognosis of patients with multiple cancers receiving immunotherapy in combination with radiotherapy [44], as well as to predict intercellular heterogeneity and prognosis in patients with advanced melanoma [45]. Su et al. utilized MRI radiomic features to construct a model that predicts the TIL level in triple-negative breast cancer (TNBC). Transcriptomic analyses subsequently confirmed that tumors with high TIL levels, as predicted by radiomics, exhibit activated immune-related pathways. High Rad-TIL tumors were found to possess a hot immune microenvironment characterized by upregulated gene markers for T-cell infiltration, cytokines, costimulators, and major histocompatibility complexes. Additionally, higher proportions of CD8^+^ T cells, follicular helper T cells, and memory B cells were observed in these tumors. This study demonstrated the feasibility of radiomic models to predict TIL status and provided a method by which TIL status can be interpreted [46]. In a research study on the use of MRI radiomic models to predict TIL levels in breast cancer, delayed-phase MRI radiomic features exhibited the most favorable predictive performance, with areas under the curve (AUCs) of 0.934 and 0.950, respectively [47]. Additionally, a study utilizing a CT and MRI radiomics-based extreme gradient boosting (XGBoost) classifier demonstrated that the levels of tumor-infiltrating CD8^+^ T cells in patients with pancreatic ductal adenocarcinoma (PDAC) could be effectively predicted. Furthermore, this approach can be implemented to identify potential patients who could benefit from immunotherapy [48, 49]. Liao et al. assessed tumor-infiltrating CD8^+^ T-cell levels in patients with hepatocellular carcinoma (HCC) by preoperative enhanced CT radiomics and found that Rad scores were positively correlated with the percentage of TILs [50].

A few studies have focused on combining radiomic features and TILs to predict tumor prognosis. Jimenez et al. developed a pretreatment prediction model involving the MRI radiomic signature and TIL level, a combination that improved the accuracy of predicting the pathological complete response to neoadjuvant systemic therapy in patients with TNBC [51]. Few studies have classified the TME based on PD-L1 expression and TILs, and radiomics can achieve precise prediction of the type of TME. Mazzaschi et al. demonstrated that 7 CT radiomic features could efficiently differentiate hot (inflammatory) from cold (desert) TME [52]. Trentini et al. showed that hot and cold tumor TME could be effectively distinguished by three CT radiomic features, with a predicted AUC of 0.94 for the hot tumor microenvironment [53].

In conclusion, radiomics studies of TILs are still evolving and have yielded some remarkable results, but the following limitations remain. (1) Most studies have focused on CD8^+^ T cells and have not classified TILs in a more detailed way. Therefore, more detailed studies on other types of TILs are needed to explore the detailed and comprehensive roles of TILs, especially B cells. (2) Current radiomics studies on TILs are from single centers and have small sample sizes. Therefore, a multicenter study with a large sample size is necessary to verify the robustness of the findings. (3) TILs should be combined with other important components of the TME to comprehensively reveal the characteristics of the TME for better prognosis assessment and clinical decision guidance.

### Other important components of the tumor microenvironment

As comprehension of the TME has gradually progressed, radiomics studies have begun to explore other vital components and cell types present within the TME. The interplay between stromal components and tumor cells is a chief instigator of tumor progression and metastasis. According to Cai et al., MRI-based radiomic features were employed to preoperatively assess the tumor-stroma ratio in rectal cancer, and they found that radiomic features outperformed the apparent diffusion coefficient in distinguishing the tumor-stroma ratio in rectal cancer [79]. Furthermore, Meng et al. utilized an XGBoost classifier based on CT and MRI radiomics to predict the tumor-stroma ratio and enhance risk stratification in patients with PDAC [80, 81]. Multiple other cell types in the TME were also included in the study. Li et al. were able to predict survival in glioma patients by establishing a preoperative T2-weighted MRI radiomic model, which could assess the extent of macrophage infiltration preoperatively [82]. Hsu et al. developed a machine learning-based MRI radiomics model to assess the enrichment levels of four immune subpopulations, including cytotoxic T lymphocytes, regulatory T cells, activated dendritic cells, and MDSCs. The radioimmunophenotype model can characterize the immune phenotype of and can predict patient prognosis [83]. Similarly, Zhang et al. used MRI-based radiomics to predict the infiltration levels of various immune cells in low-grade gliomas, including B cells, CD4^+^ T cells, CD8^+^ T cells, macrophages, neutrophils, and dendritic cells [62]. Devkota et al. developed a nanoradiomics approach for detecting the tumor response to cellular immunotherapy targeting MDSCs. Nanoradiomics revealed that TME-directed cellular immunotherapy induces subtle changes [84]. Ming et al. revealed breast cancer heterogeneity by MRI radiomics, identifying three imaging subtypes that differ in cell cycle and extracellular matrix receptor interaction pathways and cellular components, such as cancer-associated fibroblasts. These imaging subtypes have different clinical outcomes and biological features [85]. Arefan et al. predicted the abundance of 10 cell types of breast cancer, such as fibroblasts, by MRI-based radiomic features, which correlated with different aspects of TME cell type abundance [86]. Huang et al. used the CT radiomics score to predict the neutrophil–lymphocyte ratio in the TME of gastric cancer with an AUC of 0.795–0.861 [87].

Radiomics was also used to predict the expression levels of important molecules in the TME. He et al. predicted cytotoxic T-lymphocyte-associated protein 4 expression and prognosis in clear cell renal cell carcinoma (ccRCC) with CT radiomics, which helped to stratify the prognosis of ccRCC patients [88]. Gao et al. established that CT radiomic features identified PD-1 expression status and prognosis in ovarian cancer, revealing that the cause is associated with T-cell depletion [89]. Mu et al. used a deep learning approach based on PET/CT radiomics that could predict PD-L1 status as well as immunotherapy response [90]. Jiao et al. used CT radiomics to predict that interleukin-23 (IL-23) expression levels in ccRCC correlated with prognosis as well as the immune microenvironment [91]. Müller et al. developed an MRI radiomics model to predict tissue hypoxia and vascularization in mice with an AUC of 0.85 and developed a TME signature that may help to further reveal the underlying biological puzzle [92]. Perrone et al. were able to distinguish the level of inflammation in patients by CT radiomic features based on the expression of CD68 and IL-1β. Additionally, they developed and validated a radiomic model based on quantitative inflammatory features in CT images that could predict the prognosis of patients with non-small cell lung cancer (NSCLC) [93]. Wang et al. were able to effectively predict the expression level of CD27 in head and neck squamous cell carcinoma (HNSCC) patients by CT radiomics modeling [94]. Wang et al. predicted the expression profile of Ki-67 status in ovarian cancer by PET/CT radiomic features [95].

However, there have been only a limited number of studies on ultrasound radiomics exploring the important role of the TME. Mohammadi et al. evaluated the potential benefits of incorporating the tumor perimeter as a component of the TME in the quantitative analysis of ultrasound images. They demonstrated that ultrasound-based radiomics features extracted from enlarged tumor contours can differentiate between benign and malignant lymph node and breast lesions, with AUC values of 0.868 and 0.714, respectively. Their findings provide convincing evidence of the significance of the tumor periphery and the TME in the quantitative analysis of medical imaging [96].

In summary, radiomics has the capability to evaluate other crucial components of the TME. Individual or a limited number of indicators of the immune microenvironment cannot comprehensively capture the biological characteristics of the TME. Therefore, it is an emerging trend to develop a comprehensive evaluation system using multiple indicators that can comprehensively depict the characteristics of the TME. In addition, expanding the research scope of radiomics is vital. In the future, it is necessary to expand the scope and sample size of the study, integrate various indicators, and combine multiple approaches to create a comprehensive and accurate predictive model for evaluating the tumor microenvironment, which will facilitate decoding the TME and assessing cancer prognosis.

## Multiomics of radiomics, genomics, and pathomics combined to evaluate TME and predict cancer prognosis

### Radiogenomics

Radiomics is a routine method for converting images into quantitative data. Radiogenomics is a distinct application that involves the identification of radiomic features that are linked to gene expression patterns, gene mutations, and other genome-related characteristics. These radiogenomics features provide valuable insights into the underlying genetic mechanisms that contribute to disease development and progression. Radiogenomics allows for a deeper characterization and comprehensive understanding of tumor biology as well as tumor heterogeneity. Radiogenomics studies have focused on predicting and associating established biological features, such as isocitrate dehydrogenase-1 (IDH-1) [97], epidermal growth factor receptor (EGFR) [98], P53 mutation [99], BRCA1/2 [100], Kirsten rat sarcoma (KRAS) [101], BRCA1-associated protein 1 (BAP1) [102], and other gene mutations and molecular subtypes [85, 103]. Screening for imaging group biomarkers that predict gene mutations may provide diagnostic and therapeutic value for cancer intervention.

One of the applications of radiogenomics is the prediction of the mutational status of genes through radiomics. Li et al. found that deep learning-based radiomics can predict IDH1 mutation status in low-grade gliomas [104]. Jia et al. used radiomic features that can predict EGFR mutations in lung adenocarcinoma, providing a noninvasive, easy and feasible method to predict EGFR mutation status [105]. Cui et al. reported a moderate performance in the prediction of KRAS status using MRI radiomic features with an AUC of 0.72 [101]. Radiogenomics can also predict molecular subtypes of tumors based on molecular expression. Jiang et al. developed and validated MRI radiomic features to distinguish TNBC from other breast cancer subtypes. Moreover, these features can differentiate between the molecular subtypes of TNBC. Combined with TNBC transcriptomic and metabolomic data, the radiomic features demonstrated that peritumor heterogeneity is associated with immunosuppression and upregulation of fatty acid synthesis in tumors. This study further contributes to the understanding of the biological significance of radiomics [106].

Radiogenomic analysis may also be employed to decode the underlying biological mechanisms of newly identified imaging biomarkers, and classical pathway enrichment analysis can serve as a viable method for executing radiogenomic analysis. Beig et al. revealed the association of MRI radiomic features with signaling pathways for cell differentiation, cell adhesion, and angiogenesis by radiogenomic analysis, suggesting that MRI radiomic features may be significantly associated with key biological processes that influence chemotherapeutic response in glioblastoma (GBM) [107]. Jamshidi et al. performed a multilevel radiogenomic study to elucidate the MRI radiogenomic signature of GBM caused by changes in messenger RNA (mRNA) expression and DNA copy number variation (CNV). Gene set enrichment analysis (GSEA) was used to identify various oncogenic pathways with MRI signatures, identifying 34 correlations between genetic loci that showed consistent changes in gene volume and mRNA expression, leading to the construction of an MRI, mRNA and CNV radiogenomic association map [108]. Yeh et al. conducted an exhaustive radiogenomic investigation to establish correlations between radiogenomic features and 186 Kyoto Encyclopedia of Genes and Genomes pathways. Their findings revealed that all radiogenomic size characteristics were positively linked with multiple replication and proliferation pathways and negatively associated with apoptotic pathways. The gene pathways that were associated with immune system regulation and extracellular signaling exhibited the most noteworthy radiographic signature associations. The study demonstrated that breast cancer MRI radiomic features can predict underlying gene expression, suggesting the possibility that MRI radiomic features can distinguish immunologically active tumors [109]. Wu et al. devised a radiogenomics classifier to forecast three imaging subcategories, scrutinized their biological underpinnings via in-depth pathway enrichment analysis, and uncovered a correlation between malignant tumors and the aberrant regulation of immune-related and protein export pathways [110]. Feng et al. revealed hypoxia patterns and immunological features in ovarian cancer based on CT radiogenomics analysis, finding that patients with a low-risk subtype had an active immune microenvironment for reasons that may benefit from immunotherapy. They also constructed a radiogenomics model containing four features to reveal hypoxic risk status, which had AUCs of 0.900 and 0.703 in the training and test cohorts, respectively [111]. Feng et al. were able to accurately predict the macrotrabecular-massive subtype in patients with HCC using a CT radiomic model, and findings based on high-throughput and single-cell RNA sequencing revealed that the radiomic model was associated with humoral immune dysregulation involving B-cell infiltration and immunoglobulin synthesis [112]. Yu et al. used MRI radiomics to effectively predict preoperative axillary lymph node metastasis and found an association between radiomic features and tumor microenvironmental features, including immune cells, long-chain noncoding RNA, and methylation site types, revealing a potential biological basis for MRI radiomics [113].

In summary, radiogenomics enables the noninvasive correlation and prediction of gene expression by establishing associations between genes and noninvasive imaging features. This allows for the prediction and analysis of treatment and prognosis at the molecular level. However, radiogenomics also faces certain limitations. First, most radiogenomics studies are retrospective and involve small sample sizes. Thus, it is necessary to conduct larger prospective studies to identify radiogenomics associations that can be effectively applied in clinical settings in the future. Second, there is a need to integrate data from various sources, such as transcriptomics, proteomics, and metabolomics. Joint multiomics studies would facilitate the design of more valuable biomarkers, ultimately leading to a better understanding of the TME, prognostic assessment, and overall comprehensive resolution.

### Radiotranscriptomics

Radiotranscriptomics is an emerging and crucial field that investigates the correlation between radiological features derived from medical images and gene expression profiles. This research area has significant potential in cancer diagnosis, treatment planning, and prognosis assessment. Given the inherent complexity of diseases and the influence of epigenetic factors on pathogenesis, the transcriptome offers insights into sequence modifications that may not be evident at the genomic level. Transcriptomics provides a functional context for understanding the involvement of key genes and the regulatory mechanisms underlying the selective expression variation observed in disease pathogenesis. By combining transcriptomics data with imaging, a deeper understanding of the molecular intricacies of diseases can be achieved [114, 115]. Radiotranscriptomics thus emerges as a promising approach for developing noninvasive imaging biomarkers and supporting clinical decision-making.

First, numerous studies have examined the correlation between radiomics and transcriptomics, with a focus on their biological significance. For instance, Cianflone et al. conducted a study to investigate the relationship between CT imaging features and gene expression patterns in patients with ccRCC. The study successfully identified four radiogenomics patterns that exhibited a statistically significant correlation between radiogenomics features and transcriptomes [116]. Similarly, Dovrou et al. conducted a study on non-small cell lung cancer, also finding a correlation between radiogenomics features and transcriptomes. They further validated the biological relevance of these radiomic features by conducting enrichment analyses on transcriptomics-based regression models. This analysis revealed closely related biological processes and pathways, thus providing valuable radiotranscriptomics markers and models that enhance our understanding of the link between the transcriptome and phenotype in cancer [117]. Crombé et al. investigated the correlation between MRI radiomics and differential gene expression profiles in pathway analysis of soft tissue sarcomas (STSs). The study identified three reliable patient subgroups based on delta-radiomics. Group B patients exhibited increased tumor heterogeneity, necrotic signal, infiltrative margins, peritumoral edema, and peritumoral enhancement before treatment initiation. Molecular analysis revealed downregulation of natural killer cell-mediated cytotoxicity genes and upregulation of the Hedgehog and Hippo signaling pathways. This study highlights the integration of radiomics and transcriptomics in STS, suggesting that STS with extensive changes on imaging shows overactivation of suppression of proliferation and immune response [118].

Preliminary tumor molecular typing based on radiomics and transcriptomics has been addressed by several studies. Lin et al. integrated radiomics and transcriptomics analyses to provide novel insights into the molecular annotation of radiographic features and effective risk stratification in NSCLC. Three radiotranscriptomics subtypes (RTSs) were identified using radiomics and pathway enrichment profiles: RTS1 (proliferative subtype), RTS2 (metabolic subtype), and RTS3 (immune-activated subtype). RTS3 exhibited increased infiltration of the majority of immune cells. RTSs have the potential to stratify patients with molecular heterogeneity, revealing a relationship between molecular phenotypes and radiomic features [119]. In another study, Zeng et al. utilized CT radiomic features to predict gene mutations and mRNA-based molecular subtypes. They employed a combination of multiomics data (radiomics, genomics, transcriptomics, and proteomics) to predict the overall survival (OS) of patients with ccRCC, achieving a 5-year OS AUC value of 0.846. This study suggests that CT radiomics has the potential to serve as a feasible method for predicting gene mutations, molecular subtypes, and overall survival in ccRCC patients [120]. Rabasco et al. conducted a study on the molecular subtypes of locally advanced HNSCC using radiomics. They successfully improved the prognostic value of localized regional control by combining radiomic and transcriptomic features [121]. Huang et al. employed CT radiomics to differentiate between various molecular subtypes of HNSCC, including RNA-defined HPV status, five DNA methylation isoforms, four gene expression isoforms, and five somatic gene mutations. Their results demonstrated the ability of quantitative image features to differentiate between multiple molecular phenotypes [122]. In the study conducted by Le et al., transcriptome subtypes in glioblastoma patients were predicted using XGBoost-based radiomics modeling. They identified 13 radiomics features in the model that achieved high prediction accuracies for classical (70.9%), mesenchymal (73.3%), neural (88.4%), and proneural subtypes (88.4%) [123].

In recent studies, the combination of radiomics and transcriptomics has been explored. Fan et al. developed a radiotranscriptomic signature using serum miRNA levels and CT texture features to predict the response to radiotherapy in patients with NSCLC. This signature could serve as an independent biomarker for evaluating the response to radiation therapy in NSCLC patients [124]. Trivizakis et al. constructed a multiomics machine learning model that incorporated deep features and transcriptomics in an NSCLC study. The model successfully predicted molecular subtypes and histological subtypes with AUC performances of up to 0.831 and 0.925, respectively. The clinical significance of this high-performance predictive model lies in its ability to provide prognostic value and facilitate optimal therapeutic assessment [125]. Tixier et al. investigated the reflection of tumor transcriptomics through radiomics features extracted from 18F-fluorodeoxyglucose (FDG) PET images. By analyzing FDG-PET image features and transcriptomic data of patients with locally advanced head and neck cancer, they demonstrated that FDG PET radiomics features could be utilized to infer tissue gene expression and cellular pathway activity in tumors [126]. Wu et al. conducted a study that identified correlations between radiological, pathological, and molecular features of bladder cancer using multimodal data analysis, including CT, whole-slice imaging, and transcriptomics. The study identified 13 prognostically relevant features from CT and whole-slide images, and the fusion of radiologic and pathologic features achieved higher accuracy than the single-modality approach, with an AUC of 0.89. This research illustrated the potential for using multimodal data analysis in relevant clinical applications and demonstrated the correlation between CT, pathologic slides, and molecular features [127].

Despite some previous studies correlating radiomics with transcriptomics, establishing a causal relationship between the two has remained elusive. However, transcriptomics data from single cells have not yet been utilized. Thus, in future research, the integration of single-cell transcriptomics data with radiomics will lead to more precise predictions of tumor heterogeneity, microenvironments, and the efficacy and prognosis of tumor therapy.

### Radiopathomics

Pathomics is an innovative interdisciplinary field that integrates the domains of pathology and artificial intelligence. It is based on the fundamental principle of utilizing a state-of-the-art full-slide scanner to digitize full-slide images, followed by the automatic extraction and classification of histological features and the subsequent conversion of this information into binary data. The extracted features are then processed through sophisticated self-learning computer algorithms, facilitating tasks such as cancer classification and outcome prediction [128]. Pathomics enables a detailed spatial exploration of the entire tumor landscape and its most aggressive elements from standard hematoxylin and eosin slides.

The development of pathomics is relatively recent, and pathomic studies are mainly focused on predicting patient prognosis. Chen et al. established that various pathomic features derived from H&E slides are independent predictors of gastric cancer prognosis and become possible potential predictors of adjuvant chemotherapy decisions [129]. Chen et al. used machine learning-based pathological features as novel prognostic markers for patients with ccRCC [130]. Fassler et al. demonstrated that pathomics can be used as an assessment of the abundance and spatial distribution patterns of TIL infiltration, an important biomarker in breast cancer, and that peritumoral and intratumoral TIL aggregation is associated with longer survival [131].

Another important research area is the combination of radiomics and pathomics. Jimenez et al. demonstrated a possible cross-scale association between digital pathology and CT imaging that could be used to identify relevant imaging and histopathological features to accurately differentiate lung adenocarcinoma from squamous cell carcinoma [132]. Brancato et al., in a glioma study, similarly showed a possible cross-scale association between digital pathomics and MRI. This not only contributes to the understanding of GBM intratumoral heterogeneity but also provides new insights into the combined multiomics approach [133]. Feng et al. developed and validated an artificially intelligent imaging pathomics fusion model using pretreatment MRI and H&E-stained biopsy slides to predict pathologic complete response in patients with locally advanced rectal cancer, with a combined model AUC of 0.812, significantly higher than other unimodal models [134]. Wan et al. effectively predicted a good pathological response after neoadjuvant radiotherapy for locally advanced rectal cancer with a model combining radiomic and pathomic features [135]. Wang et al. developed a machine learning nomogram model consisting of pathomic features, radiomic features, immune scores, and clinical features to reliably predict postoperative overall survival and disease-free survival in patients with colorectal lung metastases [60].

The concept of “digital biopsy” can be redefined through the utilization of noninvasive technology, achieved by the optimal integration of radiomics and pathomics. Additionally, the integration of radiomics and pathomics can enhance the comprehensive characterization of tumors with heterogeneity. Future studies need to prioritize the incorporation of genomics and proteomics data to construct comprehensive tumor prediction models that encompass macroscopic radiological data alongside microscopic pathological information.

### Pathogenomics

Cao et al. proposed a deep learning model based on histopathology images to predict microsatellite status, and the AUCs of this model were 0.88 and 0.85. Notably, the model was able to identify five distinct pathology imaging features that were associated with mutation burden and DNA damage repair-related genotypes in the genome, as well as antitumor immune activation pathways in the transcriptome. The predictive model offers the possibility of multiomics association through interpretability associated with pathological, genomic and transcriptomic phenotypes [136]. Ziemys et al. demonstrated the potential of digital pathomics in transcriptional analysis combined with the spatial distribution of immune cells to effectively predict the clinical response to BRAF inhibition in metastatic melanoma [137]. Huang et al. identified N7-methylguanosine modification (m7G) in pancancer as an innovative marker for predicting clinical outcome and immunotherapy efficacy. Cellular pathway enrichment analysis showed that m7G scores were strongly associated with invasion, the cell cycle, and DNA damage and repair. In several cancers, m7G scores were positively correlated with tumor immune dysfunction and exclusion. The XGBoost-based pathomics model accurately predicted m7G scores with an AUC of 0.97. Furthermore, scRNA-seq analysis showed that m7G significantly differed between cells in the TME, indicating its potential as a therapeutic target for cancer treatment [138]. Yu et al. reported good performance in predicting survival and immunotherapy response in cervical cancer patients by pathomic characterization and genomic profiling [139]. Sammut et al. integrated digital pathology and genomic and transcriptomic data from pretreatment breast cancer samples and correlated the pathological outcomes of surgery (complete remission or residual disease) with a multiomics profile. Research has demonstrated that the response to treatment is influenced by the pretreatment tumor ecosystem, and its multiomics landscape can be incorporated into predictive models using machine learning. The degree of residual disease after treatment is linearly related to pretreatment characteristics, such as tumor mutation and copy number landscapes, tumor proliferation, immune impact, and T-cell dysfunction and rejection [140].

Pathomics, as an emerging research method, is currently in the early stages of investigation. Despite the significant potential and prospects of supplementing and enhancing histopathology with other omics data, current research is primarily limited to small-scale datasets from single institutions. Subsequent studies that offer large-scale, multimodal datasets may facilitate the development of elaborate integration strategies, further unleashing the potential of pathomics and quantitative tissue morphology.

### Multiomics combination of radiomics, pathomics and genomics in cancer prognosis

**Fig. 2:**
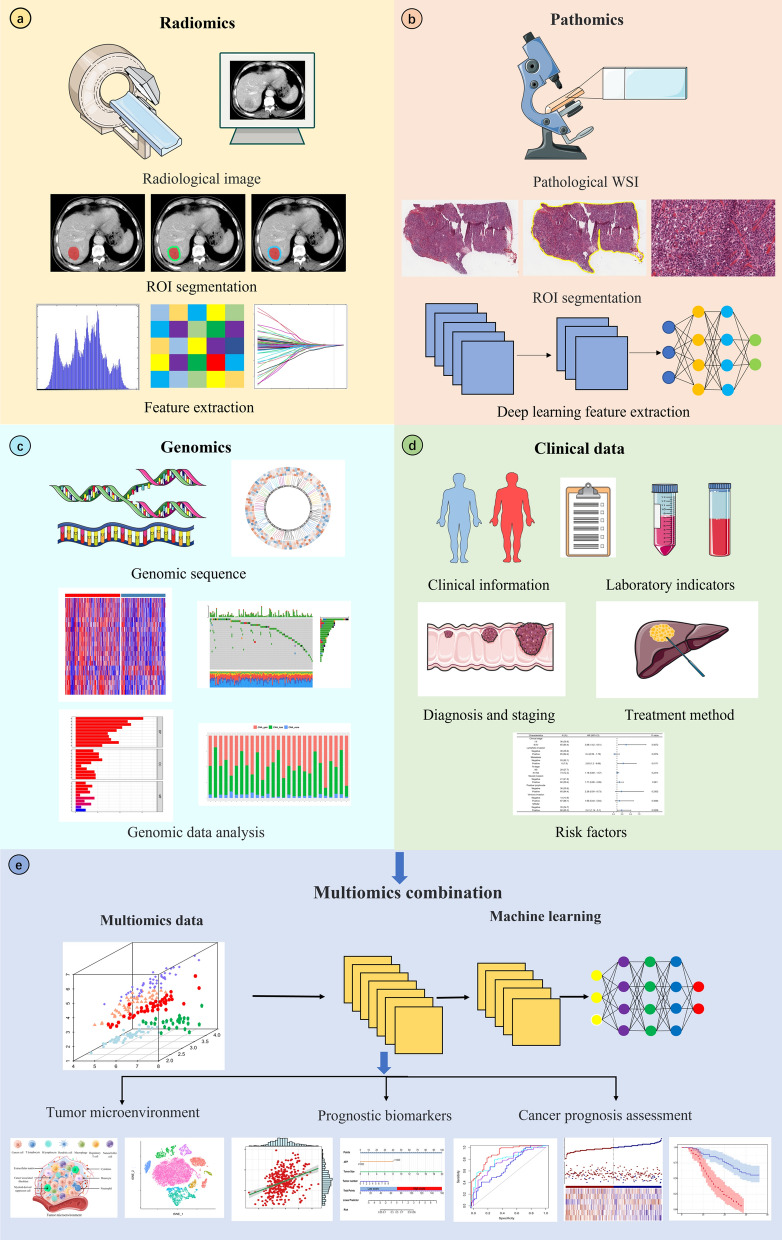
The flow chart of multiomics combination. **a** Radiomics process mainly includes image acquisition, region of interest (ROI) segmentation, radiomics feature extraction. **b** Pathomics process includes acquisition of pathological images, whole slide images (WSI) ROI segmentation, extraction features of deep learning. **c** Genomics process mainly includes acquisition of genomics data, analysis of genomics data, etc. **d** Clinical data include demographic information, laboratory examination, diagnosis and staging, treatment method, etc. **e** Multiomics combinations. Multiomics data are fused by machine learning methods to construct combinatorial models for tumor microenvironment exploration and cancer prognosis evaluation, and to develop comprehensive prognosis biomarkers

Multidimensional data from radiomics, pathomics and genomics for multiomics fusion will further comprehensively evaluate the TME and cancer prognosis (Fig. 2). However, there are currently very few studies on this area, which holds great potential for future development. Table 2 summarizes current multiomics clinical studies. Boehm et al. collected a multimodal dataset of patients with advanced high-grade plasmacytoma ovarian cancer and identified quantitative features associated with prognosis. By fusing histopathological, radiological and clinicogenomic machine learning models, they demonstrated that integration through multimodal data is a promising technique to improve the risk stratification of cancer patients [141]. Vanguri et al. evaluated the predictive power of the immunotherapy response in NSCLC by integrating radiology, histopathology, and genomic features. Machine learning was employed to synthesize the multimodal features into a risk prediction model. The study revealed that the multimodal model AUC value of 0.80 surpassed that of any single variable. These findings establish a quantitative foundation for the utilization of multimodal integration features in conjunction with machine learning to enhance the accuracy of immunotherapy response prediction in NSCLC patients [142].

**Table 2:** Radiomics-based multiomics combinations study for cancer prognosis

Tumor type	Sample size	Evaluation indicators	Multiomics approaches	Model	Training set	Test set	References
HGSOC	444	OS	Histopathological CT radiologics Genomics Clinical data	Cox model	N/A	0.61	[141]
NSCLC	247	Immunotherapy response	Pathological CT radiomics Genomics Clinical data	DyAM model	0.8	N/A	[142]
Glioma	176	OS	Pathological MRI radiomics Genomics Clinical data	DOF model	0.788	N/A	[145]

*HGSOC* high-grade serous ovarian cancer, *NSCLC* non-small cell lung cancer, *OS* overall survival, *CT* computed tomography, *MRI* magnetic resonance imaging, *DyAM* dynamic deep attention-based multiple-instance learning model with masking, *DOF* deep orthogonal fusion, *N/A* not applicable

Vaidya et al. developed a quantitative radiomics risk score and associated nomogram to predict DFS and the additional benefits of adjuvant chemotherapy after surgery for early-stage NSCLC. Radiopathomic studies have revealed that intratumoral radiomic features are correlated with various features that explain TIL-nucleus interactions. High-risk patients are predicted to have more disorganized and disordered microstructures on CT images, with corresponding whole-slide tissue images showing very dense, tightly bound spatial arrangements of cancer cell nuclei clusters. Radiogenomic features were associated with angiogenesis, proliferation, cell differentiation, T-cell and lymphocyte activation, and biological pathways of chemotaxis. Additionally, the peritumoral Haralick energy feature was inversely correlated with macrophage chemotaxis [143]. Wang et al. [144] first developed an imagingomics model to predict DFS with optimal performance in patients with locally advanced breast cancer. Then, tumor heterogeneity was analyzed by GSEA and differentially expressed gene enrichment. GSEA found that the immune-related interferon-gamma pathway was enriched in the high-scoring subgroup of the imaging set and that the cytokine‒cytokine interaction pathway was downregulated in the low-scoring subgroup. Gene Ontology enrichment analysis showed that tumors in the low-scoring subgroup differed from those in the high-scoring group in terms of cell differentiation, while tumors in the low-scoring and high-scoring subgroups had differing immune and TME, and activated natural killer cells were higher in the low-scoring group. Pathohistological studies revealed 23 histopathological features that were significantly different between the high- and low-scoring groups, with the two groups differing in cellular centrifugation, nucleus thickness and diameter. Tumors in the high-scoring group differed morphologically from those in the low-scoring group, supporting the idea that imaging histology scores reflect differences in cell differentiation. Imaging histology revealed the heterogeneity of tumor cells and the TME, genomics and pathomics explored the biological significance of imaging histological features, and the combination of the three comprehensively revealed and decoded the TME characteristics of locally advanced breast cancer and predicted prognosis [144]. Braman et al. further refined the clinical grading and molecular staging of glioma patients by developing a deep orthogonal fusion model of radiomics, pathomics, and genomics to predict the prognosis of glioblastoma patients and stratify glioma patients [145]. Table 3 summarizes the advantages and disadvantages of studies with different omics combinations.

**Table 3:** Advantages and disadvantages of different approaches in terms of TME and cancer prognosis

Methods	Advantages	Disadvantages
Radiogenomics	Investigating the biological significance of tumors, facilitating a comprehensive understanding of gene phenotypes Predicting macrolevel imaging biomarkers associated with the genome, enabling noninvasive molecular typing as well as prognostic evaluation and efficacy assessment of tumors	Small sample, single-center study Easy to overfit data in the study results Time-consuming image segmentation High cost of genomics data acquisition
Radiotranscriptomics	Exhibiting correlations, enabling exploration of biological significance Predicting tumor molecular typing and prognostic assessment and facilitates the codevelopment of novel biomarkers	Small sample size and single-center design Between transcriptomic and imaging data can show correlation but cannot establish causation Transcriptome data from current studies represent the average of cell populations, possibly failing to effectively reflect tumor heterogeneity. Addressing this issue could be achieved with single-cell RNA sequencing technology
Radiopathomics	Integrating macroradiomics and microscale pathomics enables improved characterization of tumor heterogeneity and the development of novel biomarkers This combination redefines the concept of “digital biopsy”	Image segmentation is time-consuming The biological interpretation between radiomics and pathomics features requires further investigation The combination of genomic and proteomic data is necessary to build a comprehensive predictive model for characterizing tumors from the macroscopic to microscopic level
Pathogenomics	Pathomics provides a global view of the tumor, genomics responds to the biology of the tumor, and the histological context of the genomic data is important for a comprehensive understanding of tumor heterogeneity The association of the two provides biological interpretation, and the combination of the two allows for the development of novel biomarkers and assessment of tumor prognosis in terms of treatment response	Small-sample and single-center studies The association between genomic and pathohistological data will reveal correlations but cannot determine causality Pathology image segmentation is time-consuming
Multiomics combinations based on radiomics, pathomics, and genomics	Understanding the underlying biological basis of specific quantitative imaging features Obtaining comprehensive information to visualize the spatial and molecular context of cancer Discovering new diagnostic and prognostic markers Establishing a holistic and comprehensive model and methodology to explore tumorigenesis, progression, and prognosis	High dimensionality and heterogeneity of multiomics data Difficulty in acquiring multiomics data The need for further combined proteomics, metabolomics, transcriptomics and other multiomics data for integrated and comprehensive research and exploration

In summary, the interconnections among radiomics, pathomics and genomics help to build and deepen the understanding of cancer biology and imaging features. At the same time, powerful machine learning techniques can decode the complex interactions of tumors and cancer treatments, and the understanding of the TME and cancer prognostic evaluation will be significantly enhanced by combining machine learning techniques with digital images and new methods for assessing the TME at the biomolecular level. It is also possible to combine transcriptomics, proteomics, metabolomics and other multiomics studies to comprehensively decode the complexity and heterogeneity of the TME and thus perform prognostic assessment, which will be a promising direction for future multiomics-based imaging. However, there are still many difficulties to overcome to exploit this rich and complex multiomics dataset. Decoding the dynamic TME and achieving accurate prognostic assessment will require comprehensive adoption of machine learning and network analysis techniques, which we believe will ultimately change our deep understanding of tumor biology, prognostic assessment of treatment response, and precision medicine.

## Challenges and opportunities

**Fig. 3:**
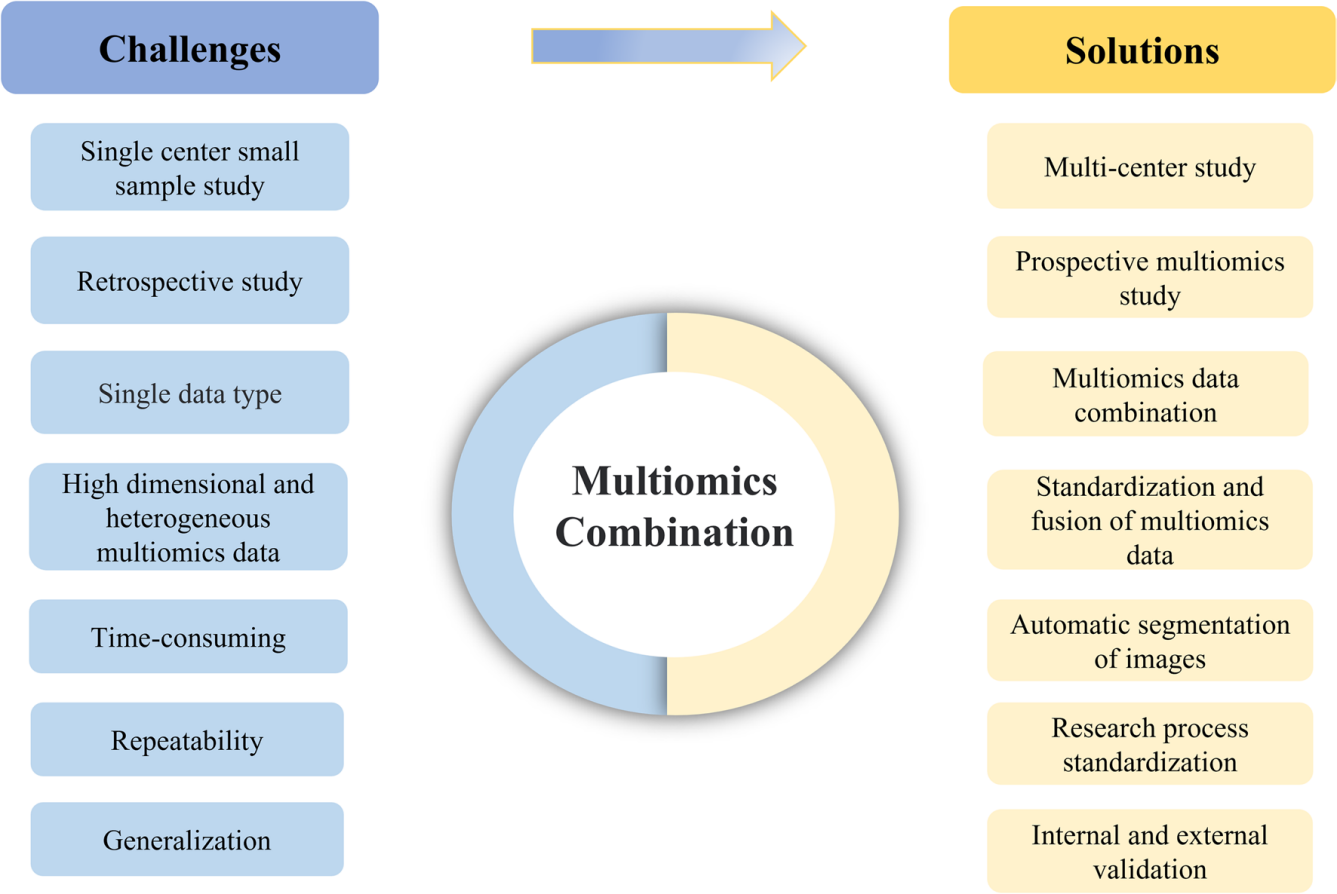
Challenges and solutions of multiomics combination. (1) The majority of current studies based on radiomics and other multiomics are small-scale, single-center, and retrospective, lacking adequate external validation. In the future, there is a need for large-scale prospective multicenter studies. (2) Presently, research on tumors predominantly focuses on single-omics studies using a singular data type. Despite the challenges in acquiring multiomics data, it is viable to collect such data through well-designed prospective studies, facilitating the integration of multimodal data for in-depth and comprehensive analysis. (3) Multiomics research faces the additional challenge of dealing with high dimensionality and heterogeneity in the data. Standardizing and fusing the multiomics data is necessary. The fusion of multiomics can be effectively achieved through machine learning techniques. (4) The processes of radiomics and pathomics are more time-consuming regarding image segmentation. Models that have a lower level of reproducibility and study results that exhibit weaker generalization. In the future, it is imperative to develop automated image segmentation methods, standardize and normalize the research process, and incorporate external validation to ensure the robustness of the findings

Figure 3 summarizes the challenges and potential solutions of multiomics combinations. Although radiomics has made some achievements in the evaluation of the TME and prognostic assessment of cancer due to its unique advantages, there are still some shortcomings and deficiencies. (1) Time-consuming and subjective nature of radiomics studies. Currently, radiomics research requires manual image segmentation, which is time consuming, and the subjectivity of manual segmentation also affects model efficacy. In future research, more efficient and reliable automatic segmentation methods should be developed, which will improve the clinical applicability, accuracy and research efficiency of radiomics and will help the further development of radiomics. (2) Lower model reproducibility. The reproducibility of current radiomics research models is relatively low, and standardized research processes are needed in the future. (3) Most radiomics-based studies are single-center and small-sample studies that lack sufficient external validation. Therefore, standardized large-scale prospective studies of patient cohorts from multiple centers are needed in the future. (4) Radiomics has a monolithic nature. For example, most current imaging histology studies assessing the TME are mainly limited to the cellular level, resulting in a single index for evaluation. Therefore, it is important to expand the scope of studies on radiomics assessment of the TME and combine other histologies into multiomics studies to integrate more indicators for the assessment of the TME and comprehensively decode its complexity and heterogeneity.

The future goal of pathomics is to integrate other omics disciplines, integrating radiomics, genomics, and transcriptomics with pathomics. Despite the potential cost of genomic and transcriptomic research, through high-throughput sequencing results, we can establish connections between the biological foundation of tumors and gather additional information about tumors and the TME. Advancements in biometric measurement technologies, such as single-cell RNA sequencing and spatial transcriptomics, allow us to gain a clearer understanding of the interactions among various subtypes of tumor cells and the dynamic TME. This will enhance our comprehension of the biological significance of tumors and the TME, further promoting the integration of genomics, transcriptomics, and other omics disciplines.

Finally, another dilemma of multiomics studies is the high dimensionality and heterogeneity of the data, and integrating quantitative measures from multimodal data for prognostic prediction is a very challenging task. Therefore, the development and implementation of multimodal fusion models in future studies requires access to matched pathology, imaging, and genomics data. Multiomics data provide a novel opportunity to comprehensively decode the TME, allowing for a more accurate and comprehensive assessment of its complexity and heterogeneity and cancer prognosis. The integration of pathological phenotypes, imaging phenotypes, and genetic phenotypes can help us (1) understand the underlying biological basis of specific quantitative imaging features, (2) obtain comprehensive information to visualize the spatial and molecular context of cancer, (3) discover new diagnostic and prognostic markers, and (4) build a holistic and comprehensive model and approach to explore and assess tumorigenesis, progression, and prognosis.

## Conclusion

In summary, the advancement of precision imaging and radiomics has introduced novel approaches for noninvasive assessment of the TME and cancer prognosis. Radiomics methodologies have already produced remarkable outcomes in TME and prognostic evaluation, and the development of machine learning and deep learning technologies will further promote the accuracy of TME as well as cancer prognostic evaluation. The multiomics combination of radiomics, pathomics and genomics will be a crucial research topic and direction for future exploration of the TME. The combination of metabolomics, transcriptomics, and proteomics will further enrich the methods for evaluating the TME and cancer prognosis. Combining imaging phenotypes with multiomics biological data can more comprehensively evaluate, characterize, and decode the TME, predict patient prognosis and further elucidate the image features and the pathophysiological and biological basis of tumor pathology. Ultimately, the establishment of new prognostic biomarkers and comprehensive prediction models will enhance the precision of diagnosis, prognosis and prediction, thereby realizing the objective of precision oncology medicine and providing optimal clinical decision support for patients’ clinical treatment and prognosis.

## Data Availability

Not applicable.

## Abbreviations

TME: Tumor microenvironment
TILs: Tumor-infiltrating lymphocytes
PD-1: Programmed cell death protein-1
PD-L1: Programmed cell death ligand-1
MDSCs: Myeloid-derived suppressor cells
VEGF: Vascular endothelial growth factor
MRI: Magnetic resonance imaging
CT: Computed tomography
TNBC: Triple-negative breast cancer
AUCs: Areas under the curve
XGBoost: Extreme gradient boosting
PDAC: Pancreatic ductal adenocarcinoma
HCC: Hepatocellular carcinoma
ccRCC: Clear cell renal cell carcinoma
IL 23: Interleukin 23
NSCLC: Non-small cell lung cancer
HNSCC: Head and neck squamous cell carcinoma
IDH-1: Isocitrate dehydrogenase-1
EGFR: Epidermal growth factor receptor
KRAS: Kirsten rat sarcoma
BAP1: BRCA1-associated protein 1
GBM: Glioblastoma
mRNA: Messenger RNA
CNV: Copy number variation
GSEA: Gene set enrichment analysis
STSs: Soft tissue sarcomas
RTSs: Radiotranscriptomics subtypes
FDG: Fluorodeoxyglucose
H&E: Hematoxylin and eosin
m7G: N7-methylguanosine modification
ROI: Region of interest
WSI: Whole slide images
UPS: Undifferentiated pleomorphic sarcomas
HGG: High-grade gliomas
BC: Breast cancer
LGG: Lower-grade gliomas
CCLM: Colorectal cancer lung metastasis
ESCC: Esophageal squamous cell carcinoma
CART: Classification and regression tree
H&E: Hematoxylin and eosin
IHC: Immunohistochemistry
LASSO: Least absolute shrinkage and selection operator
ACC: Accuracy
sPLS-DA: Sparse partial least squares discriminant analysis
RFE: Recursive feature elimination
LR: Logistic regression
GBDT: Gradient boosting decision tree
PCC: Pearson correlation coefficient
PCA: Principal component analysis
RNA-seq: RNA-sequencing
LDA: Linear discriminative analysis
SVM: Support vector machine
RF: Random forest
FACS: Fluorescence-activated cell sorting
FCM: Flow cytometry
HGSOC: High-grade serous ovarian cancer
OS: Overall survival
DyAM: Dynamic deep attention-based multiple-instance learning model with masking
DOF: Deep orthogonal fusion
N/A: Not applicable
